# Climate and Fuel Controls on North American Paleofires: Smoldering to Flaming in the Late-glacial-Holocene Transition

**DOI:** 10.1038/srep20719

**Published:** 2016-02-10

**Authors:** Y.M. Han, D.M. Peteet, R. Arimoto, J.J. Cao, Z.S. An, S. Sritrairat, B.Z. Yan

**Affiliations:** 1 SKLLQG and Key Lab of Aerosol Chemistry & Physics, Institute of Earth Environment, Chinese Academy of Sciences, Xi’an 710061, China; 2 Joint Center for Global Change Studies, Beijing 100875, China; 3 Lamont-Doherty Earth Observatory of Columbia University, Palisades, New York 10964, USA; 4 NASA/Goddard Institute for Space Studies, 2880 Broadway, NY, NY 10025; 5 Department of Environmental Science and Engineering, Xi’an Jiaotong University, Xi’an 710049, China

## Abstract

Smoldering and flaming fires, which emit different proportions of organic (OC) and black carbon (BC, in the form of char and soot), have long been recognized in modern wildfire observations but never in a paleo-record, and little is known about their interactions with climate. Here we show that in the late glacial-early Holocene transition period, when the climate was moist, relatively high quantities of char were deposited in Linsley Pond, Connecticut, USA while soot was more abundant during the warmer and drier early Holocene interval. The highest soot mass accumulation rates (MARs) occurred at the beginning of the Holocene as fuel availability increased through the climatic transition when boreal forests were locally extirpated. These variations with time are related to the different formation pathways of char and soot, which are governed by combustion efficiency. This study provides an approach for differentiating smoldering from flaming combustion in paleo-wildfire reconstructions. Our results suggest that climate and fuel loads control the occurrence of different wildfire types and precipitation may play a key role.

Biomass burning (BB) has co-evolved with the earth’s ecological system for hundreds of millions of years^1^, and it is a major global influence, affecting biogeochemical cycles, atmospheric chemistry and the carbon cycle^2^,^3^,^4^. Emissions from BB can change the composition of the atmosphere^2^, which in turn can affect climate, both regionally and globally^5^. Modern fire studies have shown that the types of emissions produced by the fires, including organic (OC), black carbon (BC), char, and soot, vary with the type of combustion (i.e., smoldering and flaming)^6^,^7^,^8^. However, smoldering and flaming fires have not been discriminated in paleo-fire records to this point, and therefore little is known about how the different wildfire types have varied with climate.

Charcoal is a macrofossil combustion residue that is widely distributed in sediments and identified microscopically while BC is produced from the incomplete combustion of biomass and fossil fuels; these two substances have been used as fire indicators for several decades^9^,^10^,^11^,^12^. The reconstructed histories of paleo-fires show pronounced spatial variability as well as clear relationships to climate^13^,^14^, but the mechanisms responsible for such differences in space and time have not been fully established.

Two kinds of BC are produced in combustion processes through different formation pathways^15^: char is a combustion residue formed directly by pyrolysis in smoldering fires while soot is a combustion condensate produced by gas-to-particle conversion at relatively high temperatures (>600 °C) in flame. Due to the different ways in which they form, the relative proportions of char and soot vary with fire type and thus have their own distinct relationships to climate. However, the separation of char from soot has rarely been applied in sediment studies^16^,^17^, much less in investigations of long-term records of paleo-fires.

Sediments from Linsley Pond, Connecticut, USA (Fig. S1) have a well-dated accelerator mass spectrometry (AMS) radiocarbon chronology (Fig. 1A), and they have been used to investigate shifts in paleoecology and paleoclimate during the last glacial-interglacial transition^18^,^19^. This background information facilitates our investigations into the interactions among climate, ecology, and selected fire indicators. In this study, we determined BC, char, soot, and charcoal concentrations using a thermal/optical method^16^,^20^,^21^, along with the mass accumulation rates (MARs) for these species in sediments from Linsley Pond. Our objective was to investigate how these parameters vary with time and how those variations are connected with climate as indicated by macrofossil and pollen data^18^.

## Results

**Figure 1:**
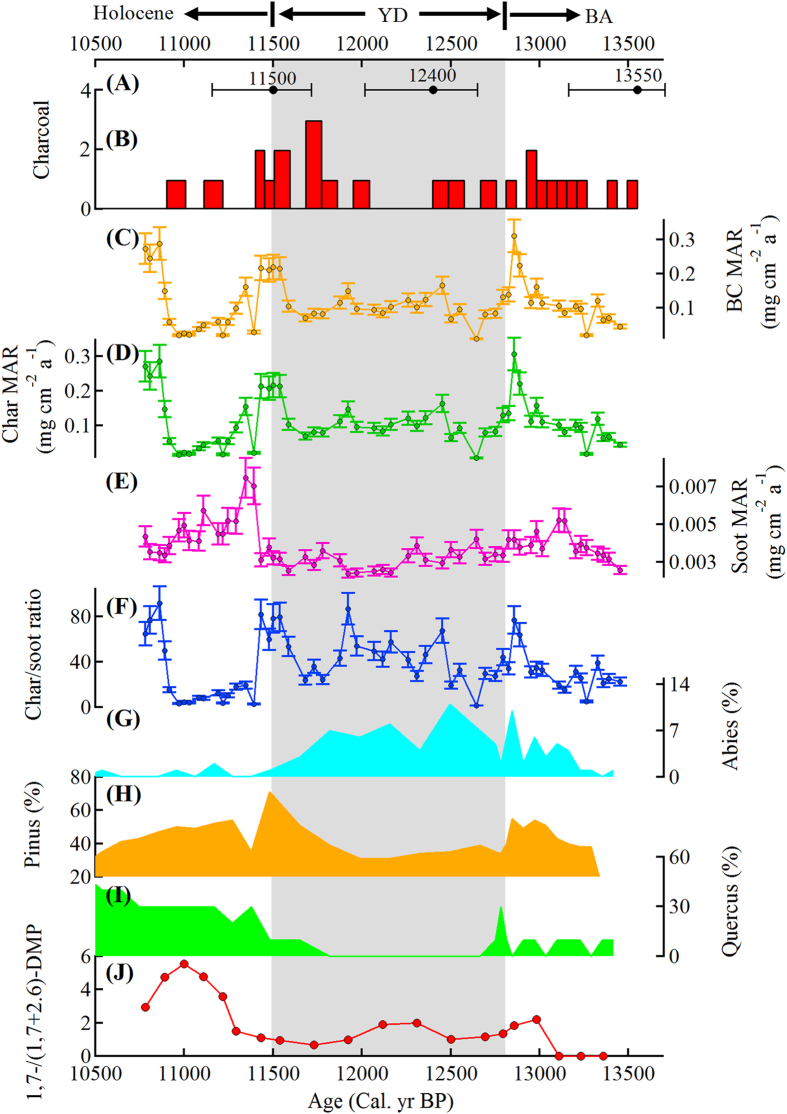
Comparison of macrocharcoal, BC, char, and soot mass accumulation rates (MARs) with local pollen and molecular compound data during the last glacial-interglacial transition at Linsley Pond, Connecticut, USA. (**A**) Calibrated radiocarbon dates with 1δ error bars; (**B**) macrofossil charcoal^18^, an indication of local fires; (**C–E**) BC, char, and soot MARs, respectively; (**F**) char/soot ratio, an indicator of the relative contribution of smoldering and flaming combustion; (**G–I**) pollen percentages from Peteet *et al*.^18^, proxies for paleoecological variation: (**G**) *Abies balsamea*, an indicator of a cool, wet climate; (**H**) *Pinus strobus.*, an indicator of a relatively dry climate; (**I**) *Quercus* spp., an indicator of warm temperatures; and (**J**) the ratio of the ratio of 1,7-dimethyl phthalate (DMP)/(1,7 + 2,6)-DMP, an indicator of the variation between softwoods and hardwoods. The shaded area indicates the Younger Dryas (YD) interval between the Holocene and the Bolling-Allerod (BA). Note: Charcoal is expressed as number per 50 cc sample and pollen data are expressed as percentages of total pollen. Errors bars show the estimated uncertainty at the 1-δ standard deviation level.

BC concentrations varied between 0.6 and 23.3 mg g^−1^ (dry weight), and char was more abundant than soot, accounting for 95% of total BC on average; this led to similar temporal variations in the BC- and char-MARs (Fig. 1C,D). The MARs for BC, char, and soot showed trends similar, but not identical, to their corresponding concentrations (correlation coefficients of 0.89−0.96, p < 0.001, Fig. S2), and this was due to the influence of sedimentation rate^22^.

Char MARs exhibited clear coupling to wet climate intervals as inferred from macrofossil and pollen data (Fig. 1). During the warm Bølling-Allerød (BA) interstadial, the climate was temperate and mesic as evidenced by the appearance of pollen of temperate *Quercus* spp. (oak) and pollen and macrofossils of fir, *Abies balsamea* (a mesic conifer)^18^. The char MARs during this time showed a slow increase and peaked at the end of the BA when temperate oak species became more abundant and boreal spruce (*Picea* spp.) declined. The colder, wet YD that followed was also characterized by high char MARs, but with some occasional troughs, and the char MARs showed a peak at the end of the YD when boreal trees, including *Larix laricina* (larch)*, Picea* and *Abies balsamea* abruptly disappeared.

The early Holocene immediately post-YD showed a sharp decrease in char MARs, and during that time, thermophilous plants, including *Quercus* spp. and *Pinus strobus* (white pine) thrived, indicating a warmer, drier climate. Indeed, the atmospheric temperature in the early Holocene increased 3–6 °C in 50 yr^18^. The decline in char MARs was followed by an abrupt increase at ~10,800 cal. yr BP (depth of 9.65 m) when the temperature was warm and *Tsuga canadensis* (eastern hemlock) reached a maximum abundance in the vicinity of Linsley Pond. Similar palynological results indicating a warm, mesic climate are typical at other sites throughout the region (Alpine Swamp, New Jersey; Sutherland Pond, New York; Minnewaska and Mohonk Lakes, New York)^23^. Overall, there were five char-MAR peaks during the studied interval; these occurred at 12,800, 12,400, 11,900, 11,500, and 10,800 cal. yr BP, and all of these correspond to mesic climate except the 11,500 peak which occurred at the climate transition point – the Holocene boundary.

Overall, the trends in the soot MAR profile were opposite those for char (Fig. 1D,E)–for example, stable and low soot MARs were found for the cold and wet YD period. An abrupt increase in soot MARs occurred in the early Holocene when an increase in *P. strobus* indicated a warmer, drier climate, and a decrease in soot MARs subsequently occurred when *P. strobus* declined, the abundance of oaks increased, and warm, more mesic conditions prevailed.

**Figure 2:**
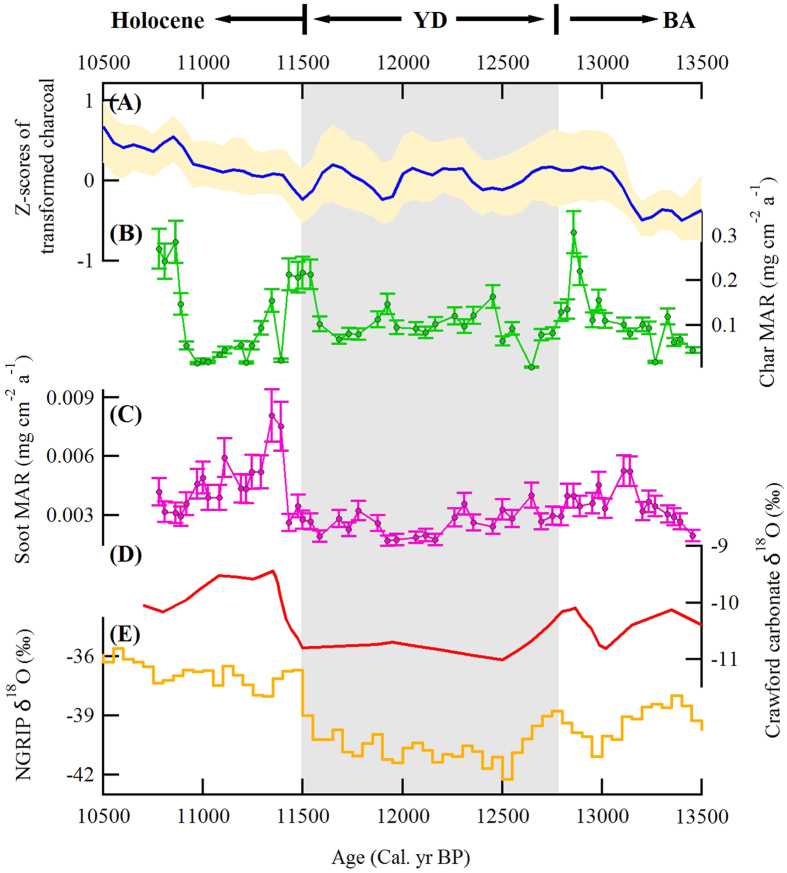
Char and soot MARs in Linsley Pond sediments in comparison with regional and global climate parameters. (**A**) Z-scores of combined and transformed charcoal data from North America^36^, an indicator of regional wildfires; (**B**) char MARs, indicating local smoldering fires; (**C**) soot MARs, indicating regional flaming fires; (**D**) carbonate δ^18^O profile from Crawford Lake in the Great Lakes region^29^, which is close to the Linsley Pond in this study, indicating the regional climate; (**E**) NGRIP δ^18^O^24^, an indicator of northern-hemisphere temperature. Shadowed area indicates the Younger Dryas (YD) interval between the Holocene and the Bolling-Allerod (BA). Errors bars show the estimated uncertainty at the 1-δ standard deviation level.

In contrast to the char MARs, which show a clear relationship with local pollen data as described above, the soot MARs exhibited a stronger relationship with regional climate. That is, the soot MAR time-series showed a clear “V” shaped pattern with lower values evident during the YD period. Furthermore, the pattern was synchronous with the low NGRIP δ^18^O^24^ and low carbonate δ^18^O at Crawford Lake, which is in the Great Lakes region, close to our study area. We note, however, that the timing for the end of YD for the NGRIP δ^18^O^24^ is not exactly consistent with the timing from northeastern America (Fig. 2E), likely due at least in part to dating uncertainties in the ice cores and radiocarbon chronology in sediments^25^.

Most notably, dramatic changes in the MARs of BC, char, and soot occurred when the climate shifted abruptly. For example, the high MARs for BC and char decreased at the ends of the BA and the YD, and a sharp increase in soot MARs occurred at the beginning of the Holocene. These findings suggest that wildfires were more prevalent when the climate was transitioning from one state to the next as has been suggested in previous studies^10^. This is reasonable because fuel availability increases when one type of vegetation dies out and is replaced by another. The high char MARs (Fig. 1D) at the end of the YD are evidence for a preponderance of smoldering combustion while the transition to the warmer and drier climate of the Holocene may have provided fuels and conditions favorable for flaming fires. Soot MARs in the early Holocene remained at relatively high levels compared with those in the YD and BA (t-test, p values < 0.01).

Macrofossil charcoal^18^, an indicator of local BB, showed no clear trends with respect to variations in climate (Fig. 1B). However, at the end of the YD there was a slight increase in charcoal as fuel increased and the climate warmed, similar to what was observed for char. Relatively little charcoal was found in the early Holocene, and this is opposite the pattern observed for soot, but consistent with the low char MARs.

## Discussion

### Smoldering and flaming fires in relation to precipitation and forest type

In modern wildfire studies, combustion efficiencies have been used to differentiate smoldering from flaming fires^26^,^27^ (see Supplementary Information): higher combustion efficiencies tend to favor flaming fires which produce more soot than char^7^. As char and soot form by different mechanisms^15^, their burial in sediments provides a means for investigating relationships between climate and fire type. Statistical analysis of global modern fire data^6^ have shown that greater precipitation results in lower combustion efficiencies (correlation coefficient of −0.52, p < 0.01 for the two variables, see Table 1 of ref. 6), and this in turn affects the relative proportions of char and soot produced by the fires^8^. A decrease of combustion efficiency from >0.92 for dry fuel to <0.86 for wet fuel also has been demonstrated in laboratory studies^28^, and this links fire type to fuel moisture and thus precipitation.

**Figure 3:**
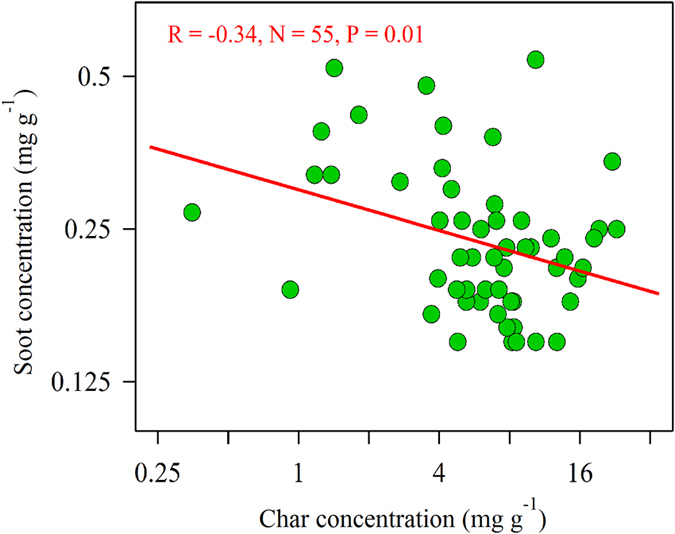
Weakly negative correlation between char and soot concentrations from sediment samples of Linsley Pond, CT, USA. Note: Char and soot concentrations were log-transferred. Due to the different factors influencing char and soot deposition in lake sediments (see text for explanations), the (weakly) negative correlation between char and soot mainly reflects climatic factors (wetness and dryness) influencing char and soot production.

The variations in char and soot MARs observed in the Linsley Pond sediments can be connected to climate-driven changes in combustion efficiency and fire type. Indeed, regional variations in temperature and moisture as recorded by lacustrine carbonate oxygen isotopes in nearby regions, including the Great Lakes^29^ co-vary with the soot MARs (Fig. 2C,D): high soot MARs during the BA and early Holocene occurred when the climate was warmer and drier compared with lower values in the cold, wetter YD. An abrupt increase in soot MARs and sharp decreases in the char MARs occurred at the beginning of the Holocene (Fig. 1D,E), and this was concurrent with (1) increases in temperature and a drier climate^18^ both locally and regionally, (2) shallower water in lakes regionally, and (3) a decrease in organic matter in the adjacent Sky Lakes, New York region^23^. Furthermore, there was a weak but significant anti-correlation between char and soot concentrations in the Linsley Pond sediments (r = 0.34, p = 0.01; Fig. 3), which is consistent with the variations in flaming and smoldering emissions caused by different climate conditions.

We note that in addition to the climate factors that influence the proportions of char and soot, the deposition of char and soot in lake sediments also can be impacted by several other factors, including differences in transport pathways^15^,^30^,^31^, lake depositional processes, and the simultaneous occurrence of different wildfire types. With reference to the fire type, smoldering and flaming combustion can transition between types during a given fire event. In general, however, large char particles (although smaller than charcoal in general) tend to be deposited near field while fine soot particles are more regionally dispersed^15^,^32^,^33^. Thus, it should be kept in mind that in spite of the weak anti-correlation between char and soot found in our study (Fig. 3), this might not be the case for other environments because of possible impacts from small local fires. Nevertheless, the weak anti-correlations of char and soot shown in Fig. 3 imply that differences in climate favor one type of fire over the other.

To investigate whether the char and soot production were related to combustion efficiency^6^, we calculated the char/soot ratios for the sediments and examined the data for possible relationships with precipitation. We note that in most cases the trends in char/soot ratios were similar to those of the char-MARs (Fig. 1D,F), and this is consistent with the fact that smoldering fires are dominant in rain forests^34^. Some small peaks in the char/soot ratios that were not accompanied by char-MAR peaks occurred during mesic conditions as indicated by pollen data. For example, a char/soot ratio peak occurred at ~12,200 cal. BP, and this corresponds with a peak of *Abies balsamea*, a mesic climate indicator.

Modern wildfire observations suggest that the fuel loads may affect wildfire emissions^6^, however, this depends in complex ways on climate as well as the types of vegetation and soils. In this regard, dramatic shifts in forest type were seen at the end of the YD when boreal trees (*Picea* spp.*, Larix* spp.*, Betula papyrifera;* paper birch) died out and temperate trees (*Pinus strobus, Quercus* spp.) replaced them^18^. During the early Holocene, char and soot MARs showed opposing patterns, and the relative abundances of a polycyclic aromatic hydrocarbon marker ((1,7 dimethyl phthalate DMP)/(1,7 DMP + 2,6 DMP), Fig. 1J) indicated that there was a shift from softwoods (shown to be *Pinus strobus*) to hardwoods (shown to be *Quercus* spp.).

### Paleofire signals-effects of scale

Differences in the transport properties of char and soot have long been suspected because soot particles are typically sub-micrometer in size and more readily transported than the super-micrometer char particles^15^,^31^. However, due to the lack of methods for differentiating between char and soot^20^, it has only recently been confirmed through studies of aerosols and sediments that soot transport tends to be regional in nature while char is more local^30^,^33^. Along these lines, Jeong *et al*.^33^ found that soot concentrations in PM_2.5_ (particulate matter with an aerodynamic diameters ≤2.5 μm) were similar for both urban and rural areas in North America while char concentrations were considerably higher in urban areas. Comparative studies of lake sediment covering the past 150 years showed similar historical profiles for soot from different sites compared with more variable profiles for char^22^,^30^. Thus, we suggest that for sediment studies such as ours, soot may best be regarded as an indicator of regional flaming combustion while char is more of an indicator of local smoldering fires.

The argument for a soot/regional flaming combustion connection is bolstered by the high injection altitude normally associated with flaming combustion^35^ because that promotes the broad dispersal of the burning-emitted particles. In comparison, the larger char particles are more representative of local smoldering fires and more local deposition. The latter suggestion is supported by wildfire reconstructions which showed that high char MARs and charcoal concentrations at the end of the BA (Fig. 1A,C) occurred at the same time as high combined Z scores for charcoal data from Northern America^36^ (Fig. 2A). We note that in some cases, char may also indicate fires at a broader scale; for example, the 10,800 char-MAR peak corresponds well with the regional mesic climate as implied by fossil pollen from the Great Lakes region^29^ but not so much with the local pollen data (Fig. 1G–I).

### Implications concerning fire emissions and spatial heterogeneity

The BC variations in the Linsley Pond sediments were synchronous with char (Fig. 1C,D) because char was the dominant fraction of total BC (average 95%). More interesting is the fact that the percentage of char was far higher than that found in semi-arid areas^16^ where annual precipitation is ~400–600 mm compared with **~**1200 mm at Linsley Pond^18^ (Table S1). This finding is consistent with a study by Ward *et al*.^34^ who found that smoldering fires were dominant in rain forests while the Brazilian savanna cerrado was more susceptible to flaming fires.

Neither charcoal nor total BC can be used to distinguish between the types of paleofires, and perhaps as a consequence, the history of paleofires reconstructed from charcoal and BC at times have varied greatly^14^,^37^. Explanations for such discrepancies include differences in the sources for the two materials as well as in their transport processes and distance of the sources from the point of deposition^37^. A history of biomass burning in Northern America reconstructed from 35 charcoal records showed no systematic relationship with climate^36^, and it presents a totally different profile from the soot MARs obtained in the present study (Fig. 2A,C).

Little attention so far has been paid to how combustion intensity (or efficiency) influences char and charcoal production. The macrofossil charcoal profile from Linsley Pond shows some similarities with char and BC, but is not highly correlative. Previous studies have reported that charcoal-based reconstructions of paleofires may be associated with either wet^38^ or dry^39^ conditions, but at least in principle, one would expect that the prevailing climate would play a role in determining the characteristics of the fires and the types of substances they emit^40^. The ability to distinguish between soot and char has enabled us to go beyond a simple reconstruction of paleofire occurrences and instead consider some relationships between climate and fire types and their emissions.

BC is strongly light absorbing, and as a consequence, the particles emitted by fires are generally agreed to be important in terms of the Earth’s radiative budget^41^,^42^. It is beyond the scope of this study to estimate the impacts of the paleofires in this regard, but the results suggest that a greater production of soot occurs in dry climate zones and more char in wetter areas. This means that the types of fires and possibly even their potential environmental effects may be predicted from models using climate variables. In any case, further studies of char and soot will provide a better understanding of climate/fire relationships as more information is obtained from studies of other sedimentary materials and emissions from modern fires.

## Methods

### Sampling

Linsley Pond is a small kettle lake in southern Connecticut (41°18′ N, 72°45′ W, 65 m above sea level) located in the headwaters of the Branford River (Fig. S1). A 12-m sediment core was retrieved from the ice-covered pond using a modified Livingstone piston corer in about 9.2 m of water, and the chronology (for the depth of 9.5–12 m) was reconstructed in 1993 using accelerator mass spectrometric dating^18^. Despite the uncertainties in the AMS dating at that time, it was the data for paleoecologically-significant, dated macrofossils coupled with pollen stratigraphy^18^ that clearly proved a Younger Dryas (YD) cooling in northeastern North America. Those data very clearly show a characteristic pattern for the region, which included a warming Allerod with the increase in oaks that then disappeared as the climate cooled during the YD and boreal trees (spruce, fir, paper birch, alder) increased. White pines increased at the Holocene boundary, and this signified warmth and drought. Linear interpolation was used to reconstruct the calibrated age (cal. yr. BP) from the AMS ^14^C-dates^43^.

## Experimental

In 2009, the sediment core was sub-sampled continuously at 2–3 cm intervals from the depth of 11.53 to 9.50 m, which covers a period spanning the last glacial-to-interglacial transition. BC, char, and soot were measured using a thermal optical method following Han *et al*.^20^,^21^. In brief, samples of ~0.15 g were pretreated with acids to remove carbonates, minerals, metal oxides, and ions, and they were then filtered through pre-fired quartz filters. The carbonaceous particles captured on the filters were analyzed following the IMPROVE protocols. Stepwise heating produced different carbon fractions, i.e. four organic carbon fractions OC1, OC2, OC3, and OC4 at 120, 240, 450, 550 °C in helium atmosphere, and three elemental carbon (EC, the traditional term used in aerosol studies and is exchangeable with black carbon, BC, which is commonly used in sediment and soil science^44^) fractions, EC1, EC2, EC3 at 550, 750, and 850 °C in a 98% helium/2% oxygen atmosphere. Meanwhile, pyrolized carbon (PC) was produced in the inert helium atmosphere, and it was monitored by measuring the return of the laser reflectance to its initial value. Total BC (traditionally it also called EC in aerosol studies) was defined as the sum of EC fractions minus PC according to the IMPROVE protocol, while EC1 minus PC was defined as char and the sum of EC2 and EC3 as soot according to Han *et al*.^20^. The distributional homogeneity of residues on the quartz filter after chemical pretreatment has been tested in our previous studies by replicate analysis^21^,^30^,^45^. The carbon analyzer was calibrated with known quantities of methane daily. Replicate analyses were performed at the rate of one per group of 10 samples.

Polycyclic aromatic hydrocarbons (PAHs) are produced from BB, and they are used as BB markers, especially some of the high-molecular-weight congeners. Here, the ratio of 1,7-dimethylphenanthrane (DMP) to 1,7-DMP plus 2,6-DMP [1,7/(1,7 + 2,6)-DMP] was used to indicate the shift between softwood and hardwood burning. The method for the measurement of PAHs is described in Yan *et al*.^46^. Briefly, the sediments were transferred to ashed aluminum trays and then dried in an oven at 40 °C under a flow of air filtered through Florisil until dryness. The dried sediments were weighed, ground, and then extracted using 70:30 DCM:MeOH with the use of an Accelerated Solvent Extraction system 200 (ASE 200, previous Dionex now Thermo Fisher). A 100 ppm 17β(H),21β(H)-Hopane (Sigma Aldrich Cat. No. 07562) was added to the extraction cell before extraction. Extracts were evaporated to about 1 mL under a gentle flow of N_2_, then an alumina gel liquid chromatograph column was used to purify hydrocarbons. Samples were concentrated to ~200 mL, then transferred to GC vials and spiked with 0.5 ppm dueterated PAH standard mix (Supelco, Cat. No. 48418). PAH analysis was performed on a Varian GC-MS (now Agilent) with an option of large volume injection to achieve a lower detection limit.

### Mass accumulation rate calculation for BC, char and soot

Mass accumulation rate (MAR, mg cm^−2^ a^−1^) for BC, char, and soot was calculated using the formula below:


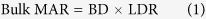



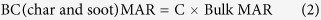


where C (mg g^−1^) is the concentration of BC, char, or soot; BD (g cm^−3^) represents the bulk density of each sample, which was measured by the dry weight of bulk samples in a 1-cm^3^ cubic container; and LDR (cm a^−1^) is the deposition rate, which is computed following the formula:


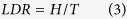


where H (cm) represents the thickness of the sampled layer, and T (a) is the duration of its deposition.

### Uncertainty estimation for BC, char, and soot MAR

The uncertainties for mass accumulation rates (MARs) of BC, char, and soot stem from two sources: the uncertainties associated with (1) the concentration measurements and (2) the MAR calculations. These overall uncertainties are calculated as the sum of the relative errors of the two parts.

Uncertainties in the estimation of the BC, char, and soot concentrations were calculated using the formula below following Han *et al*.^16^:


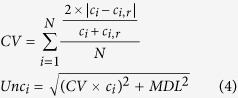


Where CV = coefficient of variance; N = number of samples; Ci = concentration of initial analysis; Ci,r = concentration of sample “i” replicate analysis; Unc_i_ = uncertainty; MDL = minimum detection limit. The average CV for BC, char, and soot from the replicate analyses (one per ten samples) was used as the common CV for the corresponding carbon content in this study. The MDL of the DRI Model 2001 carbon analyzers is 0.06 μg cm^−2^ for BC, 0.06 μg cm^−2^ for char and 0.03 μg cm^−2^ for soot, respectively^16^. The calculated uncertainties were all lower than 10% for BC, and lower than 14% for char and soot.

Uncertainty for MAR calculation including the age error estimation and the density measurement. The age errors are estimated based on 1-δ measurement error. The errors for the bulk density measurement are directly estimated as ~2%, which come mainly from the operation and container. The uncertainty errors were presented in the figures following Tierney *et al*.^47^.

## Additional Information

**How to cite this article**: Han, Y.M. *et al*. Climate and Fuel Controls on North American Paleofires: Smoldering to Flaming in the Late-glacial-Holocene Transition. *Sci. Rep.* **6**, 20719; doi: 10.1038/srep20719 (2016).

## Supplementary Material

Supplementary Information
