# Supplementary material for: Climate and Fuel Controls on North American Paleofires: Smoldering to Flaming in the Late-glacial-Holocene Transition
Source: Sci Rep. 2016 Feb 10;6:20719. doi: 10.1038/srep20719 (PMC4748283; doi:10.1038/srep20719)
Supplement: Supplementary Information [file srep20719-s1.pdf]

Supplementary Online materials

Climate and Fuel Controls on North American Paleofires:

Flaming and Smoldering in the Late-glacial-Holocene Transition

Y.M. Han *, D. Peteet, R. Arimoto, J.J. Cao, Z.S. An,

S. Sritrairat, B.Z. Yan

* To whom correspondences should be addressed. E-mail:

yongming@ieecas.cn (Y.M. Han)

This file is 10 pages in total, including materials listed below:

Background and Experimental;

Figures S1-S2;

Table S1.

Background and materials

1. Background

A detailed description of the sedimentary record of Linsley Pond, Connecticut, USA (Fig. S1) has been presented previously [1,2]. Connecticut has a maritime climate, with both continental and oceanic features, and three types of air masses typically affect this region: (1) cold, dry subarctic air in winter, (2) moist, warm air from the Gulf of Mexico and the subtropical Atlantic, and (3) moist maritime air from the North Atlantic. Year-round average temperatures range from ~5 to ~16°C, and annual precipitation is 1100−1200 mm [2]. The vegetation of the region transitions from hemlock-white pine-hardwood forests in the north and deciduous forests in the south[1] where oak-chestnut forests dominate, and other species include *Acer saccharum* (sugar maple), *Carya* (hickory), *Fagus grandifolia* (beech), *Betula lutea* (yellow birch), *Pinus strobus and Tsuga canadensis* (hemlock). Paleoecological data indicate that the region experienced a warming, moist climate during the Bolling-Allerod and a cold, wetter climate during the Younger-Dryas period. Warm, dry conditions prevailed in the early Holocene [1], and that was followed by moister conditions and an increase in hemlocks.

Linsley Pond (Fig. S1), at an elevation 65 m, is a small kettle lake in southern Connecticut (41° 18' N, 72° 45' W). Located in the headwaters region of the Branford River in North Branford, CT., it is strongly eutrophic, ~10 hectares in area, 14.8 m deep and extensively studied [1]. A previously collected sediment core was used in this study and the chronology for the depth of 9.5-12 m was published in 1993 [1].

2. Differences between flaming and smoldering combustion and the substances they emit

Combustion typically proceeds through a sequence of stages, namely ignition, flaming, smoldering, and extinction; and different types of emissions are produced during each stage [3,4]. In general, the ignition and extinction stages are comparatively short in duration and produce few emissions; therefore, the emissions from a given fire are largely determined by the balance between flaming and smoldering combustion. Indeed, flaming and smoldering combustions are typically thought of as the two main fire types, and these are related to the combustion efficiency (CE) or modified combustion efficiency (MCE) of the fire. The latter is much easier for measurement than CE, and it is defined as the ratio of measured emitted CO_2 to the sum of CO and CO_2 as follows:

$$\triangle CO_2/(\triangle CO_2 + \triangle CO)$$

For a pure flaming fire, the MCE is near 0.99 while for smoldering fires MCEs are lower, between 0.65-0.85 and most often ~0.80. Flaming and smoldering combustion can simultaneously occur in a single fire event, and there may be no distinct transitional state between types. An overall fire-integrated MCE near 0.9 indicates roughly equal amounts of biomass consumption by flaming and smoldering processes. The different combustion processes can produce varying amounts of CO_2 and CO, as well as oxidized and reduced compounds, and these are represented by their emission factors [5]. Flaming combustion primarily emits highly oxidized compounds, such as CO_2, NOx, and SO_2; and fires of this type also favor the production of soot. Smoldering fires, with low MCEs, produce more char, CO, CH_4, non-methane organic compounds, primary OC, etc [4,6].

3. Validation of BC, char, and soot measurements and applications to sediment studies

Black carbon is not single chemical component but a “combustion continuum”

[7-9], varying from partially charred material to highly condensed soot. Nonetheless, most commonly used methods measure total BC, but there is still no universally accepted method for determining BC concentrations [10], and the problem that results is that the data for BC are method dependent [11]. The popular methods can produce a difference of BC concentration for a given sediment sample up to 571 times [12]. Apart from the misidentification of BC and artifacts involving non-BC species, the quantification of different parts of BC by different methods also contributes to the uncertainties in the BC data that have been reported [10,13].

Thermal optical method[14-16] [16,17] have become the most popular approach for the quantification of BC in aerosol studies (BC is also referred to as elemental carbon, EC, in aerosol science), and the different variations of this method can produce results for a given samples that vary by a factor of two [14]. Although this method has the disadvantage of sometimes measuring non-BC coals as BC [10,18], it has offsetting advantages, such as charring corrections with lasers [15] and relatively low uncertainty [14], and as a result it has become wide used, with the analysis of over 100,000 aerosol samples documented [19]. We adapted this method [20,21] to bridge the gap between the quantification of BC for aerosol and soil and sediment samples, with the intent of facilitating comparisons between the different types of media [22]. This required the development of a chemical pretreatment step to remove the interferences from mineral dusts and metal oxides that affect the BC determinations [10,20].

The ability to distinguish char from soot was further advanced by comparing thermograms of pure char and soot standard reference materials (SRMs) using the IMPROVE (Interagency Monitoring of Protected Visual Environments) protocol [23]. That developmental work showed that char and soot SRMs evolved in different stages of the analytical process. Comparisons of the IMPROVE soot with the CTO-375 BC

method [22], which is supposed to specifically measure soot carbon [13,24,25], demonstrated comparable concentrations and good correlations between the two methods. Thermograms of the IMPROVE soot were similar to those of the CTO-375 method coupled with quantification by the IMPROVE protocol[23]. The validation of the IMPROVE method for BC quantification in soil and sediments also has been validated by stepwise incremental additions of SRMs [26].

Techniques to differentiate char from soot have been used in aerosol studies to demonstrate that the two substances have the different transport mechanisms [27,28], and the transport pathways of different aerosol plumes [29] have shown good correlations between char and levoglucosan, HULIS (humic-like substances) and potassium ions [29-31], which are widely used as source markers of biomass burning. Char/soot ratios also have been used as source markers due to variations in the proportions of char and soot emitted by different sources [30-32]. One hundred-fifty year histories of soot concentrations is sediment cores from Daihai and Chaohu Lakes [22], which are about 150 km from each other, showed strong similarities, and the patterns observed reflected the regional dispersion of soot [9] caused by industrialization in eastern China. That also helped validate the use of the IMPROVE protocol for differentiating char from soot. A stronger correlation of polycyclic aromatic hydrocarbons (PAHs) with soot than with char from environmental sediment and soil samples [33] is consistent with the suggestion of a higher absorption capacity of soot compared with char based on studies with SRMs [34]. Also, char and soot concentrations from sediments in Daihai Lake clearly showed an increase in biomass burning during the Holocene that was attributed to human activities in north China [35].

References:

1 Peteet, D. M. *et al.* Late-Glacial Pollen, Macrofossils and Fish Remains in Northeastern USA - the Younger Dryas Oscillation. *Quaternary Sci. Rev.* **12**, 597-612 (1993).
2 Shemesh, A. & Peteet, D. Oxygen isotopes in fresh water biogenic opal - Northeastern US Allerod-Younger Dryas temperature shift. *Geophys. Res. Lett.* **25**, 1935-1938, doi:10.1029/98gl01443 (1998).
3 Yokelson, R. J., Susott, R., Ward, D. E., Reardon, J. & Griffith, D. W. T. Emissions from smoldering combustion of biomass measured by open-path Fourier transform infrared spectroscopy. *J. Geophys. Res.* **102**, 18865-18877, doi:10.1029/97jd00852 (1997).
4 van Leeuwen, T. T. & van der Werf, G. R. Spatial and temporal variability in the ratio of trace gases emitted from biomass burning. *Atmos. Chem. Phys.* **11**, 3611-3629, doi:10.5194/acp-11-3611-2011 (2011).
5 Yokelson, R. J. *et al.* Trace gas measurements in nascent, aged, and cloud-processed smoke from African savanna fires by airborne Fourier transform infrared spectroscopy (AFTIR). *J. Geophys. Res.* **108**, doi:8478 10.1029/2002jd002322 (2003).
6 Christian, T. J. *et al.* Comprehensive laboratory measurements of biomass-burning emissions: 2. First intercomparison of open-path FTIR, PTR-MS, and GC- MS/FID/ECD. *J. Geophys. Res.* **109**, doi:D02311 10.1029/2003jd003874 (2004).
7 Goldberg, E. D. *Black carbon in the environment*. (John Wiley & Sons, Inc., 1985).
8 Hedges, J. I. *et al.* The molecularly-uncharacterized component of nonliving organic matter in natural environments. *Org. Geochem.* **31**, 945-958 (2000).
9 Masiello, C. A. New directions in black carbon organic geochemistry. *Mar. Chem.* **92**, 201-213, doi:10.1016/j.marchem.2004.06.043 (2004).
10 Hammes, K. *et al.* Comparison of quantification methods to measure fire-derived (black/elemental) carbon in soils and sediments using reference materials from soil, water, sediment and the atmosphere. *Global Biogeochem. Cycles* **21**, doi:Gb3016 10.1029/2006gb002914 (2007).
11 Lim, B. & Cachier, H. Determination of black carbon by chemical oxidation and thermal treatment in recent marine and lake sediments and Cretaceous-Tertiary clays. *Chem. Geol.* **131**, 143-154 (1996).
12 Schmidt, M. W. I. *et al.* Comparative analysis of black carbon in soils. *Global Biogeochem. Cycles* **15**, 163-167 (2001).
13 Elmquist, M., Cornelissen, G., Kukulska, Z. & Gustafsson, O. Distinct oxidative stabilities of char versus soot black carbon: Implications for quantification and environmental recalcitrance. *Global Biogeochem. Cycles* **20**, GB2009, doi:2010.1029/2005GB002629, doi:10.1029/2005gb002629 (2006).
14 Watson, J. G., Chow, J. C. & Chen, L.-W. A. Summary of organic and elemental carbon/black carbon analysis methods and intercomparisons. *J. Aero. Air Qual. Res.* **5**, 65-102 (2005).
15 Chow, J. C. *et al.* The dri thermal/optical reflectance carbon analysis system: description, evaluation and applications in U.S. Air quality studies. *Atmospheric Environment. Part A. General Topics* **27**, 1185-1201 (1993).
16 Birch, M. E. & Cary, R. A. Elemental carbon-based method for monitoring occupational exposures to particulate diesel exhaust. *Aerosol Sci. Tech.* **25**, 221-241 (1996).
17 Cavalli, F., Viana, M., Yttri, K. E., Genberg, J. & Putaud, J. P. Toward a standardised thermal-optical protocol for measuring atmospheric organic and elemental carbon: the EUSAAR protocol. *Atmospheric Measurement Techniques* **3**, 79-89 (2010).
18 Han, Y. M. *et al.* The effect of acidification on the determination of elemental carbon, char-, and soot-elemental carbon in soils and sediments. *Chemosphere* **75**, 92-99, doi:10.1016/j.chemosphere.2008.11.044 (2009).
19 Chow, J. C., Watson, J. G., Crow, D., Lowenthal, D. H. & Merrifield, T. Comparison of IMPROVE and NIOSH carbon measurements. *Aerosol Sci. Tech.* **34**, 23-34 (2001).
20 Han, Y. N. *et al.* Evaluation of the thermal/optical reflectance method for quantification of elemental carbon in sediments. *Chemosphere* **69**, 526-533, doi:10.1016/j.chemosphere.2007.03.035 (2007).
21 Husain, L. *et al.* Trends in atmospheric elemental carbon concentrations from 1835 to 2005. *J. Geophys. Res.* **113**, doi:10.1029/2007JD009398 (2008).
22 Han, Y. M. *et al.* Comparison of elemental carbon in lake sediments measured by TOR, TOT and CTO methods and 150-year pollution history in Eastern China. *Environ. Sci. Technol.* **45**, 5287-5293 (2011).

23 Han, Y. M. *et al.* Evaluation of the thermal/optical reflectance method for discrimination between char- and soot-EC. *Chemosphere* **69**, 569-574, doi:10.1016/j.chemosphere.2007.03.024 (2007).
24 Gustafsson, O. & Gschwend, P. M. in *Molecular Markers in Environmental Geochemistry* Vol. 671 *ACS Symposium Series* 365-381 (1997).
25 Gustafsson, O. *et al.* Evaluation of a protocol for the quantification of black carbon in sediments. *Global Biogeochem. Cycles* **15**, 881-890 (2001).
26 Zhan, C. *et al.* Validation and application of a thermal-optical reflectance (TOR) method for measuring black carbon in loess sediments. *Chemosphere* **91**, 1462-1470, doi:10.1016/j.chemosphere.2012.12.011 (2013).
27 Han, Y. M., Lee, S. C., Cao, J. J., Ho, K. F. & An, Z. S. Spatial distribution and seasonal variation of char-EC and soot-EC in the atmosphere over China. *Atmos. Environ.* **43**, 6066-6073, doi::10.1016/j.atmosenv.2009.08.018 (2009).
28 Jeong, C.-H. *et al.* Identification of the Sources and Geographic Origins of Black Carbon using Factor Analysis at Paired Rural and Urban sites. *Environ. Sci. Technol.* **47**, 8462-8470, doi:10.1021/es304695t (2013).
29 Lim, S. *et al.* Ionic and carbonaceous compositions of PM10, PM2.5 and PM1.0 at Gosan ABC Superstation and their ratios as source signature. *Atmos. Chem. Phys.* **12**, 2007-2024, doi:10.5194/acp-12-2007-2012 (2012).
30 Minoura, H., Morikawa, T., Mizohata, A. & Sakamoto, K. Carbonaceous aerosol and its characteristics observed in Tokyo and south Kanto region. *Atmos. Environ.* **61**, 605-613, doi:10.1016/j.atmosenv.2012.07.058 (2012).
31 Chuang, M.-T. *et al.* Characterization of aerosol chemical properties from near-source biomass burning in the northern Indochina during 7-SEAS/Dongsha experiment. *Atmos. Environ.* **78**, 72-81, doi:10.1016/j.atmosenv.2012.06.056 (2013).
32 Han, Y. M., Cao, J. J., Lee, S. C., Ho, K. F. & An, Z. S. Different characteristics of char and soot in the atmosphere and their ratio as an indicator for source identification in Xi'an, China. *Atmos. Chem. Phys.* **10**, 595-607 (2010).
33 Han, Y. M. *et al.* Stronger association of polycyclic aromatic hydrocarbons with soot than with char in soils and sediments. *Chemosphere* **119**, 1335-1345 (2015).
34 Cornelissen, G. *et al.* Extensive sorption of organic compounds to black carbon, coal, and kerogen in sediments and soils: Mechanisms and consequences for distribution, bioaccumulation, and biodegradation. *Environ. Sci. Technol.* **39**, 6881-6895, doi:10.1021/es050191b (2005).
35 Han, Y. M., Marlon, J., Cao, J. J., Jin, Z. D. & An, Z. S. Holocene linkages between char, soot, biomass burning and climate from Lake Daihai, China. *Global Biogeochem. Cycles* **26**, doi:10.1029/2012GB004413 (2012).
36 Han, Y. M. *et al.* Elemental carbon and polycyclic aromatic compounds in a 150-yr sediment core from Lake Qinghai, Tibetan Plateau, China: Influence of regional and local sources and transport pathways. *Environ. Sci. Technol.*, DOI: 10.1021/es504568m (2015).
37 Zhan, C. *et al.* Spatial distributions and sequestrations of organic carbon and black carbon in soils from the Chinese loess plateau. *Sci. Total Environ.* **465**, 255-266, doi:10.1016/j.scitotenv.2012.10.113 (2013).

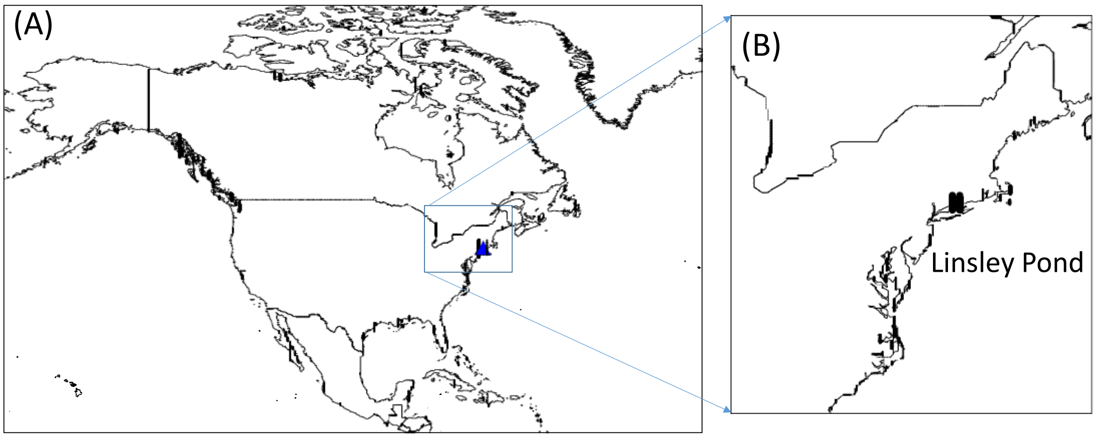

Fig. S1. Location of Linsley Pond, Connecticut, USA. Both figures were created with the software of QGIS version 2.12.0 (Open Source Geospatial Foundation Project, http://qgis.osgeo.org). The data for the figures were downloaded from Natural Earth (www.naturalearthdata.com).

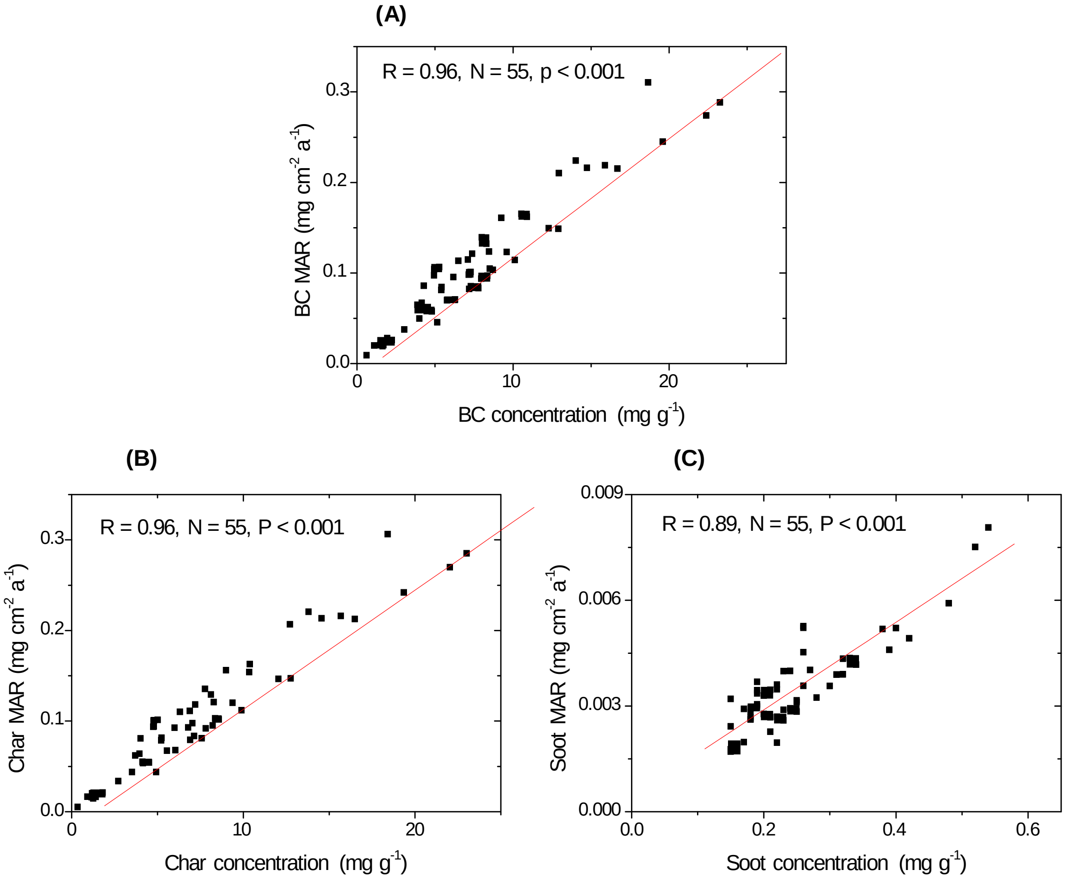

Fig. S2. Correlations between concentrations and mass accumulation rates (MARs) for BC, char, and soot. (A) BC concentration vs. BC MAR; (B) Char concentration vs. char MAR ; and (C) Soot concentration vs. soot MAR.

Table S1. Black carbon (BC) parameters in Linsley Pond, USA compared with other sites. The concentrations and ratios are expressed as ranges and (average).

Location	Precipitation[1] (mm)	Temperature[1] (°C)	Period	BC ($mg\ g^{-1}$)	char ($mg\ g^{-1}$)	soot ($mg\ g^{-1}$)	Char/soot	References
Linsley Pond, Eastern America	1100-1200	5-16	Last glacial-Holocene transition	0.62-23.26 (8.05)	0.35-23.01 (7.80)	0.15-0.54 (0.25)	1.28-91.66 (35.89)	This study
Daihai, Northern China	350-450	5.1	Holocene	0.10-6.14 (1.67)	0.04-5.79 (1.47)	0.06-0.43 (0.20)	0.59-25.51 (7.18)	35
Chaohu, Eastern China	1032-1205	16	the past 150 yr	0.61-2.03 (1.13)	0.48-1.58 (0.93)	0.08-0.47 (0.20)	2.82-8.02 (5.14)	22
Taihu, Eastern China (Industrialized region)	1180	15-17	the past 150 yr	0.41-1.95 (1.01)	0.01-1.43 (0.60)	0.31-1.09 (0.42)	0.03-4.21 (1.51)	22
Qinghai, Western China	324-413	0.3-1.1	the past 150 yr	0.40-1.45 (1.57)	0.18-1.09 (0.30)	0.22-0.35 (0.26)	0.63-3.30 (1.16)	36
Chinese Loess Plateau	185-750	3.6-14.3	Quaternary and modern (surface soil)	0.02-5.50 (0.65)	0.003-4.19 (0.47)	0.01-1.32 (0.18)	0.48-17.27 (3.18)	37

[1]Annual precipitation and temperature.
